# Trainee educational curriculum to standardize central venous catheter repair

**DOI:** 10.1186/s12909-023-04977-9

**Published:** 2023-12-19

**Authors:** Marina Reppucci, Connor Prendergast, Katherine Flynn, Sharon Scarbro, S. Christopher Derderian, Jose Diaz-Miron

**Affiliations:** 1 Department of General Surgery, The Mount Sinai Hospital, New York, NY USA; 2 Pediatric Surgery, Children’s Hospital Colorado, 13213 E 16th Ave, Box 323, 80045 Aurora, CO USA; 3 Adult and Child Consortium for Health Outcomes Research and Delivery Science, University of Colorado Anschutz Medical Campus, Aurora, CO USA

**Keywords:** Central venous catheter, Pediatric Surgery, Central venous catheter repair

## Abstract

**Introduction:**

Children may require durable central venous catheters (CVCs) for various reasons. CVC-related integrity complications are common and can often be repaired at the bedside to increase lifetime. Variability in repair techniques can lead to complications, including the need for repeat repair and Central Line Associated Blood Stream Infection (CLABSI).

**Methods:**

The impact of an educational curriculum to standardize tunneled CVC repairs for trainees on a pediatric surgery service was studied, focusing on comfort level with tunneled CVC repair and to determine the impact on complication rates. Rotating trainees studied a dedicated audiovisual educational curriculum comprised of a video, educational slides, and a practical component from November 2020 through January 2022. Experience and comfort level with tunneled CVC repairs were assessed before and after the rotation. CVCs repaired during the duration of the study were evaluated and compared to the period prior.

**Results:**

Forty-nine individuals completed the pre- and post-training survey. Respondents (34.7%, n = 17) most commonly reported one year of surgical experience, and (79.6%, n = 39) had never observed or assisted in a repair previously. Following training, respondents felt more comfortable with all aspects of the CVC repair process (p < 0.001). There were no statistically significant differences in re-repair rates or CLABSI rates following the implementation of the curriculum.

**Conclusions:**

Tunneled CVC procedural repair variability can be standardized with a dedicated educational curriculum for rotating trainees, which improves knowledge and comfort with such procedures.

**Supplementary Information:**

The online version contains supplementary material available at 10.1186/s12909-023-04977-9.

## Introduction

Central venous catheters (CVC) are essential for the treatment of several clinical conditions, including in children who require long-term parenteral nutrition, chemotherapy, hydration, additional medications, and frequent blood testing [1]. Though CVCs are widely used, they are not without complications. These include less frequently encountered infections, such as central line-associated bloodstream infection (CLABSI) and exit-site infection, or more commonly, mechanical complications, such as occlusion, dislodgement, and rupture of the line [2, 3]. These complications may result in hospitalization, premature removal of the line, and increased healthcare costs. In the setting of mechanical complications, specifically fracture or rupture of the external portion of the line, repairs can be performed at the bedside to increase its longevity and prevent removal and replacement, and are associated with no increase incidence of infection rate but eliminates the risks of anesthesia associated with CVC replacement in the operating room [4, 5].

Variability in repair techniques can lead to increased complications and there is currently no consensus on the appropriate approach to bedside repairs of tunneled CVC lines. There has been substantial emphasis on quality improvement initiatives to standardize care within medicine, and standardization of practices has been shown to improve outcomes for other pediatric surgery problems [6–8]. The pediatric surgery service at Children’s Hospital Colorado (CHCO) is responsible for tunneled CVC troubleshooting and repairs. Similar to many subspecialty surgical disciplines at teaching hospitals, this service is staffed by rotating general surgery residents. Despite experience with clinical care and surgical technique, trainees have varying levels of experience with pediatric surgery-specific problems, such as tunneled CVC repairs. This was seen as an opportunity for standardization and education.

Simulation-based training has become an essential component of surgical training proven to teach skills [9, 10]. Multiple studies have shown that simulation-based training improves procedural knowledge and comfort levels in a variety of surgical contexts [11–13]. There is substantial evidence of the impact of simulation in the adult literature, but simulation-based training in pediatric surgery for general surgery residents remains in its infancy [14]. The primary purpose of this study was to assess the impact of a standardized education curriculum for tunneled CVC repairs on procedural comfort level for trainees rotating on a pediatric surgery service. The secondary aim was to determine if this education impacted complication rates.

## Methods

### Study design

This was a retrospective observational study conducted at CHCO. Children’s Hospital Colorado is a quaternary, freestanding, regional referral children’s hospital. All trainees who participated in the tunneled CVC repair education curriculum between November 2020 and January 2022 were asked to complete pre- and post-curriculum anonymous surveys. Those who completed both were included in this study. Trainees who did not complete both surveys were excluded. This study was reviewed and approved by the Colorado Multiple Institutional Review Board (COMIRB). All methods were carried out in accordance with our COMIRB regulations. Informed consent to participate was waived by Colorado Multiple Institutional Review Board (COMIRB), due to retrospective nature of the study.

### Intervention

An education curriculum for bedside repairs of tunneled CVCs was implemented in November 2020 to standardize the process of CVC repairs. All trainees at any level of postgraduate training who rotated on the pediatric surgery service at CHCO as well as advanced practice providers (APPs) covering non-surgical services were asked to complete an anonymous survey detailing their demographic data and experience and comfort levels with CVC repair at the start of the rotation. They subsequently completed an education curriculum, which consisted of audiovisual materials including a video (https://vimeo.com/517348712), educational slides, and a practical training component taught by a senior APP. Following completion of the curriculum, a second anonymous survey was sent to assess for changes in comfort levels with tunneled CVC repair and measure the impact of the education curriculum.

The education curriculum consists of three components: (1) a practical simulation component using a mannequin (Chester Chest, VATA, Canby, OR) and materials used in tunneled CVC repair where the trainee undergoes simulation with a senior APP that walks them through all steps of the repair process. (2) A computer-generated educational video simulating the tunneled CVC repair process. The video includes narrated information on indications for repair, appropriate set-up, the actual repair process (identifying the line defect, transecting proximally, and repairing using a commercially available tunneled CVC repair kit), and information on troubleshooting. (3) Educational slides provided in print and available for the trainees in our secure shared online drive included similar information to the video. Since our number of tunneled CVC repairs in the 2-year period before implementation was relatively low (2.6 average per month), throughout the rotation we ensured participants received continued proctoring for CVC repair procedures by an experienced member of our pediatric surgical team that included APPs, pediatric surgery fellows, and pediatric surgery attendings.

To track the primary outcome of this study (self-reported comfort with aspects of the tunneled CVC repair process), pre- and post-curriculum surveys were used. The set of questions addressed specific components of the repair process and respondents answered on a Likert scale (0–10), with 0 indicating feeling completely unqualified and 10 indicating feeling completely comfortable with performing that aspect of the repair process without supervision.

The secondary outcomes of interest were re-repair rates and CLABSI within 30 days (based on standard surgical complication tracking timeframe) of bedside tunneled CVC repair. An electronic medical record query to assess all central venous catheter repairs by the pediatric surgery service since November 2018 was performed. The pre-education period was defined as November 2018 through October 2020, and the post-education period was defined as November 2020 to January 2022. A retrospective chart review was performed to determine if the repair was performed by an APP or trainee and the subsequent post-graduate level (PGY). Re-repair was defined as any tunneled CVC that required repeat repair within 30 days of the initial re-repair. CLABSI was defined as a CLABSI within 30 days of repair of the CVC.

### Statistical analysis

Measures were reported as mean [standard deviation (SD)] for continuous variables and number (%) for categorical variables. We used the Wilcoxon signed-rank test to test for differences between pre- and post-curriculum. We performed the Wilcoxon rank-sum test on the difference between pre- and post-curriculum measures to test if there was a difference by experience (1 year vs. > 1 year). We tested for differences in re-repair and CLABSI rates pre- and post-curriculum by using logistic regression. Statistical Process Control (SPC) chart was generated to evaluate overall CLABSI rates during the study timeframe and the impact of the instituted educational curriculum. An interaction of time (before or after November 2020) by who completed the repair (residents 1–2 yrs, residents > 2 yrs, APP) was included in the model to look for a difference over time in rates.

## Results

### Overall Population

There were 59 individuals who completed the pre-curriculum survey and 50 individuals who completed the post-curriculum survey. Of these, 49 completed both surveys and were thus included in the analysis. The mean (SD) age was 31.1 (3.9) years and 55.1% [27] were female. Most trainees were from the University of Colorado and 18.4% [9] were from outside institutions. Five (10.2%) were APPs from CHCO rotating from non-surgical services, such as the inpatient medical service. Approximately half (51.0%, n = 25 had 1–2 years of training and 84.1% (37) of residents were categorical general surgery. Six (13.6%) of the residents planned to pursue pediatric surgery following training. The most common post-training plan was general surgery subspecialty (56.8%, n = 25) (Table 1).

**Table 1:** Demographic and training data of overall population**—**Data reported as mean (SD) or frequency (%). *Variables reported as proportion of residents included in study (n = 44). SD: Standard deviation; UCH: University of Colorado Hospital; OSH: Outside Hospital; APP: Advanced Practice Provider; CHCO: Children’s Hospital Colorado

	Overall Population (n = 49)
**Age**, mean (SD)	31.1 (3.9)
**Gender**, female	27 (55.1%)
**Institution Training**	
UCH	35 (71.4%)
OSH	9 (18.4%)
Rotating APP from CHCO	5 (10.2%)
**Number of Years in Training**	
1–2	25 (51.0%)
3–5	19 (38.8%)
6+	5 (10.2%)
**Residents’ Current Training Program** (n = 44)*	
General Surgery Categorical Resident	37 (84.1%)
Preliminary Surgery Resident	4 (9.1%)
Pediatric Surgery Fellow	3 (6.8%)
**Residents’ Post Training Plans** (n = 44)*	
Pediatric Surgery	6 (13.6%)
General Surgery Subspeciality (non-Pediatric Surgery)	25 (56.8%)
General Surgery Practice	9 (20.5%)
Non-General Surgery Specialty	2 (4.5%
Non-Surgical Specialty	2 (4.5%)

### Prior experience

Most individuals had no prior pediatric surgery experience (57.1%, n = 28) or had spent 3–4 weeks on a pediatric surgery service (28.6%, n = 14). Only 9 (18.8%) of individuals had previously assisted or observed a tunneled CVC repair and only 4 (8.1%) had performed a repair. Full data on prior experience is provided in Table 2.

**Table 2:** Information on prior pediatric surgery and CVC repair exposure and experience**—***Data reported as frequency (%)*

	Overall Population (n = 49)
**Time Spent on Pediatric Surgery Service Previously**	
None	28 (57.1%)
3–4 weeks	14 (28.6%)
5–8 weeks	2 (4.1%)
9 + weeks	5 (10.2%)
**Number of Central Lines Repairs Assisted or Observed Previously**	
None	39 (81.3%)
1–2	7 (14.6%)
3+	2 (4.2%)
Not Reported	1
**Number of Central Lines Repairs Performed Previously**	
None	45 (91.8%)
1–2	3 (6.1%)
3+	1 (2.0%)

### Impact of Curriculum

**Fig. 1:**
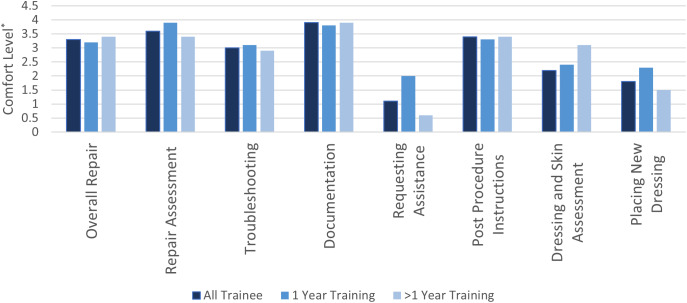
Difference in Mean Comfort Levels Changes with tunneled CVC Repair Process Pre- and Post-Education Curriculum, by Number of Years Training. ^*^Comfort level was reported on a scale of 0–10

When comparing mean comfort levels with components of the CVC repair process, there was a statistically significant increase for all 8 components addressed by the survey (Table 3). The differences in comfort levels prior to and after the curriculum were compared based on years of training. There was no statistically significant difference in comfort levels between individuals with one year of training and those with more than one year of training (Fig. 1), suggesting the curriculum impact of the curriculum may not depend on training level.

**Table 3:** Mean comfort levels with tunneled CVC repair process components before and after standardized educational curriculum**—***Data reported as mean (SD)*

“How would you rate your overall level of comfort in…”	Before	After	*p*-value
**…personally performing a central line repair procedure?**	2.2 (2.7)	5.5 (2.8)	< 0.001
**…assessing if a central line is appropriate for repair?**	2.6 (2.6)	6.2 (2.9)	< 0.001
**…troubleshooting central line catheters that are not flushing easily after repair?**	2.5 (2.5)	5.5 (2.9)	< 0.001
**…documenting central line procedures?**	3.3 (3.1)	7.1 (2.9)	< 0.001
**…requesting assistance for central line procedures?**	7.4 (3.2)	8.5 (2.6)	0.001
**…providing post procedure instructions and follow up information to patients, caregivers or provider teams following central line repair?**	3.1 (3.0)	6.5 (2.8)	< 0.001
**…assessing the dressing or skin around the line during a central line repair?**	5.9 (3.4)	8.0 (2.4)	< 0.001
**…placing a new sterile dressing around the line after a central line repair?**	6.3 (3.7)	8.0 (3.1)	< 0.001

### Secondary outcomes

**Fig. 2:**
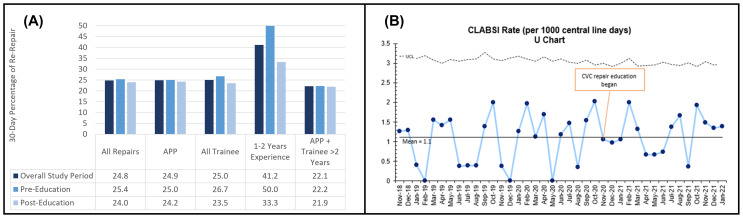
30-Day Outcomes. (**A**) 30-Day re-repair, based on individual performing repair. (**B**) Statistical process control chart for 30-day central line associated blood stream infection during study timeframe without noted differences in CLABSI rates before and after intervention

There were 113 lines repaired during the study period: 63 prior to the implementation of the education curriculum and 50 after. The rate of 30-day re-repairs was 24.8% (28) overall; it was 25.4% [16] prior to the curriculum and 24.0% [12] after the curriculum was implemented. Rates of re-repair are displayed in Fig. 2A. Differences in re-repair rates based on who performed the repair were minimal, except for trainees with 1–2 years of experience. The rate of re-repair was 50.0% prior to the curriculum and 33.3% after the curriculum. However, these differences were not statistically significant.

The overall rate of CLABSI rate during the study timeframe was 1.11 per 1000 central line days (116 infections in 104,294 central line days during our timeframe). Out of the 113 lines that required repair during the duration of the study, there were three (4.8%) CLABSIs prior to the education curriculum and five (10.0%) after resulting in pre-curriculum and post-curriculum. There were no statistically significant differences in CLABSI rates following the institution of our educational curriculum in November 2020, as noted in our SPC chart (Fig. 2B).

## Discussion

This study indicates an educational curriculum dedicated to standardizing the CVC repair process improves comfort levels in all aspects of the repair procedure. This trend was seen with all trainees who participated, regardless of their level of training. This indicates that a practical curriculum to standardize pediatric surgery procedures can benefit not just junior residents, but also senior residents and fellows. However, this curriculum did not statistically impact re-repair or CLABSI rates in our limited series.

Simulation-based education has been extensively studied in graduate medical education for both surgical and non-surgical trainees [15]. Proponents of simulation-based training argue that practicing simulations before performing procedures on patients improves patient safety. This has been confirmed by multiple studies of the impact of simulation on the performance of surgical residents and subspecialty procedures [16–20]. It has been shown to be most impactful for less commonly performed [19]. Within our study cohort, most trainees had never seen nor performed a CVC repair previously, indicating their low rates of exposure. This highlights the importance of practical education curriculums to increase familiarity with procedures prior to performing them on patients.

The implementation of a curriculum to teach trainees rotating on pediatric surgery resulted in increased confidence with all aspects of the repair process, regardless of the level of the trainee. Our findings are consistent with multiple prior studies that report improvements in confidence levels for trainees following simulation-based training [13, 14, 18, 21]. There is, however, mixed data on the impact of such curriculums on year of training. The impact of simulation training on confidence levels based on training status may be dependent upon the procedure addressed, as prior interventions have led to an increased comfort level with procedures even among faculty [22]. We did not perform a sensitivity analysis by discreet year of training, but we did not see any difference between the mean difference in confidence levels when comparing trainees with less than one year of training and those with more. Though not statistically significant, certain domains resulted in greater increases for trainees with more experience, indicating there may also be utility in tailoring education curriculums based on the experience level of the learner.

The Accreditation Council for Graduate Medical Education (ACGME) requires general surgery residents to complete at least 20 pediatric surgery cases during residency [23]. The number of pediatric surgery rotations are often minimal with variable caseloads, which highlights the heterogeneity in exposure to pediatric surgery and associated pathology and cases [14, 24]. The minimal time on pediatric surgery service, diversity of cases, and low case volume can leave trainees ill-prepared when rotating on pediatric surgery services. Simulation-based learning has been shown to be successful in improving the performance of pediatric surgeons [25]. However, most simulation models have been developed for pediatric surgery fellows, leaving a need and opportunity for educational curriculums for general surgery residents. Curriculums, such as ours, provide instruction and training on procedures that are rare for general surgery residents, but a common procedure in pediatric surgery, and can serve as a model for other procedures and education initiatives.

The secondary aim of this study was to determine if the education curriculum impacts complications associated with CVC repairs. There has been substantial research on rates of CVC complications [1–3, 26]. Most prior research does not account for bedside repairs and focuses on initially placed lines, but rates of mechanical complications, such as fracture, and kinking, have been reported to be between 10% and 18% [1, 2, 26]. Our rate of re-repair, which includes mechanical complication as well as any other indication for bedside repair, was 25%. It is possible our rate is higher than previously reported rates given the repair processes and longevity of the line may inherently increase complication rates. CLABSIs are another outcome of interest and occur at a rate of 0.7 to 7.4 per 1000 catheter days [27]. Again, these rates are based on initial placement and do not account for bedside repairs. Consistent with prior data, our reported rate during the study timeframe was 1.11 per 1000 central line days. Although outside of the scope of this study, repaired lines were categorized based on demographics including age at the time of repair, sex, race, ethnicity, weight at the time of repair, and diameter of line. No statistical differences were noted for any of these demographics and our tracked complications, including re-repairs and overall CLABSI rates.

This study is not without limitations. First, the long-term implications of this intervention are unknown. For example, though individuals report increased comfort levels with CVC repairs, it is unclear if this will be retained. Additionally, our sample size is small and represents the experience of residents rotating at a single institution, therefore, our findings may not be generalizable to all populations. Our follow-up period may also have limited our ability to detect any differences in complication rates, and longer-term analysis is warranted. Finally, consistent with any survey-based research is the concern for reporting bias and the Hawthorn effect, which could have impacted results.

## Conclusion

In conclusion, an education curriculum that targets the CVC repair process results in increased comfort and confidence with all aspects of the process for surgical trainees rotating on a pediatric surgery service, regardless of training level. However, this limited series failed to statistically affect overall CLABSI or re-repair rates. Such findings support initiatives to incorporate simulation and multi-modal education processes in pediatric surgical training or for dedicated vascular access teams to supplement learning. This curriculum can serve as a model for other low-occurrence procedures to improve trainee comfort levels and performance.

### Electronic supplementary material

Below is the link to the electronic supplementary material.

**Supplementary Material 1:** Central line repair follow-up survey

**Supplementary Material 2:** Central line repair survey

**Supplementary Material 3:** Central venous catheter (CVC) repair policy

## Data Availability

The data generated or analyzed during the current study are available from the corresponding author on reasonable request.

## Abbreviations

APP: Advanced practice providers
CHCO: Children’s Hospital Colorado
CLABSI: Central line associated blood stream infection
COMIRB: Colorado Multiple Institutional Review Board
CVC: Central venous catheter
SD: Standard deviation
