# Supplementary material for: Trainee educational curriculum to standardize central venous catheter repair
Source: BMC Med Educ. 2023 Dec 19;23:978. doi: 10.1186/s12909-023-04977-9 (PMC10731855; doi:10.1186/s12909-023-04977-9)
Supplement: Supplementary file 2 — Supplementary Material 2: Central line repair survey [file 12909_2023_4977_MOESM2_ESM.pdf]

Central Line Repair Survey

Dear participant,

Please complete this brief survey so we may better assess the experiences before and after your time on our service.

I sincerely appreciate your help with this study.

Please enter today's date	________________________________
Please list your first name initial AND your last name initial	________________________________<br>(Example: John Smith = JS)
Please select the description that applies to you	◯ Trainee at the University<br>◯ Trainee OUTSIDE of the University<br>◯ Rotating Advanced Practice Provider
Please state your current surgical training program	◯ General Surgery categorical resident<br>◯ Preliminary Surgery resident<br>◯ Pediatric Surgery fellow
Please state your plans after completing this current training program	◯ Pediatric Surgery fellowship training<br>◯ General Surgery Subspecialty training NOT including Pediatric Surgery<br>◯ Pediatric Surgery practice<br>◯ General Surgery practice<br>◯ General Surgery Subspecialty practice NOT including Pediatric Surgery<br>◯ Continued training in General Surgery<br>◯ Continued training in a Surgical discipline outside of General Surgery (Ortho, Plastics, Ob/Gyn, Urology, Neurosurgery, Ophthalmology, ENT, etc)<br>◯ Continued training in a NON-Surgical discipline (Radiology, Dermatology, Internal Medicine, Pediatrics, Neurology, etc)
Please select the number of years you have been in training (for trainees) or out in practice (for advanced practice providers)	◯ 1 year<br>◯ 2 years<br>◯ 3 years<br>◯ 4 years<br>◯ 5 years<br>◯ 6 years<br>◯ 7 years<br>◯ 8 years<br>◯ 9 years<br>◯ 10 or more years
Please state your age	________________________________
Please state your gender	◯ Male<br>◯ Female
Is this the first time you rotate on Pediatric Surgery?	◯ Yes<br>◯ No

If this is not the first time you rotate on Pediatric Surgery, please estimate the number of weeks you have PREVIOUSLY spent on a Pediatric Surgery service	◯ 1-2 weeks<br>◯ 3-4 weeks<br>◯ 5-8 weeks<br>◯ 9-12 weeks<br>◯ 13-24 weeks<br>◯ More than 24 weeks
Please estimate the number of central line (Broviac) repair procedures you have assisted or observed BEFORE starting on the Pediatric Surgery service	◯ None<br>◯ 1<br>◯ 2<br>◯ 3-5<br>◯ 6-10<br>◯ More than 10
Please estimate the number of central line (Broviac) repair procedures you have personally performed BEFORE starting on the Pediatric Surgery service	◯ None<br>◯ 1<br>◯ 2<br>◯ 3-5<br>◯ 6-10<br>◯ More than 10
If you have repaired central venous (Broviac) catheters in the past, please describe the HIGHEST LEVEL of training you received for your FIRST central line repair procedure<br><br>Independent training includes reading the instructions on the repair kit, searching for online written instructions, and searching for educational videos<br><br>Formal training includes PowerPoint presentations, written instructions, and educational videos by the procedure team where you performed your FIRST central line repair	◯ Level 1: No training at all<br>◯ Level 2: Independent training<br>◯ Level 3: Formal training<br>◯ Level 4: Formal training AND Independent training<br>◯ Level 5: Proctored procedure by another experienced provider<br>◯ Level 6: Proctored procedure by another experienced provider AND Formal training<br>◯ Level 7: Proctored procedure by another experienced provider AND Formal training AND Independent training
If you selected independent training, please check all that apply	☐ Read instructions on the repair kit package<br>☐ Searched for written instructions<br>☐ Searched for instructional videos<br>☐ Other____
Please state what other independent training you received	__________________________________
If you received formal training, please select all that apply	☐ PowerPoint presented or distributed by a proceduralist<br>☐ Written instructions presented or distributed by a proceduralist<br>☐ Educational video produced by the procedure team<br>☐ Other____
Please state what other formal training you received	__________________________________

Please use the following scale to answer the next set of questions:

0 I feel completely unqualified or highly apprehensive about this.

10 I feel completely comfortable and/or require no supervision about this.

On a scale of 0-10, how would you rate your overall level of comfort in PERSONALLY PERFORMING a central line (Broviac) repair procedure?

◯ 0
◯ 1
◯ 2
◯ 3
◯ 4
◯ 5
◯ 6
◯ 7
◯ 8
◯ 9
◯ 10

On a scale of 0-10, how would you rate your overall level of comfort in ASSESSING IF A CENTRAL LINE (BROVIAC) IS APPROPRIATE FOR REPAIR

◯ 0
◯ 1
◯ 2
◯ 3
◯ 4
◯ 5
◯ 6
◯ 7
◯ 8
◯ 9
◯ 10

On a scale of 0-10, how would you rate your overall level of comfort in TROUBLESHOOTING central line (Broviac) catheters that are not flushing easily after the repair

◯ 0
◯ 1
◯ 2
◯ 3
◯ 4
◯ 5
◯ 6
◯ 7
◯ 8
◯ 9
◯ 10

On a scale of 0-10, how would you rate your overall level of comfort in DOCUMENTING central line (Broviac) procedures

◯ 0
◯ 1
◯ 2
◯ 3
◯ 4
◯ 5
◯ 6
◯ 7
◯ 8
◯ 9
◯ 10

On a scale of 0-10, how would you rate your overall level of comfort in REQUESTING ASSISTANCE for central line (Broviac) procedures	◯ 0<br>◯ 1<br>◯ 2<br>◯ 3<br>◯ 4<br>◯ 5<br>◯ 6<br>◯ 7<br>◯ 8<br>◯ 9<br>◯ 10
On a scale of 0-10, how would you rate your overall level of comfort in providing POST PROCEDURE INSTRUCTIONS AND FOLLOW UP INFORMATION TO PATIENTS, CAREGIVERS OR PROVIDER TEAMS following central line (Broviac) repair procedures	◯ 0<br>◯ 1<br>◯ 2<br>◯ 3<br>◯ 4<br>◯ 5<br>◯ 6<br>◯ 7<br>◯ 8<br>◯ 9<br>◯ 10
On a scale of 0-10, how would you rate your overall level of comfort in ASSESSING THE DRESSING OR SKIN AROUND THE LINE during a central line (Broviac) repair procedure	◯ 0<br>◯ 1<br>◯ 2<br>◯ 3<br>◯ 4<br>◯ 5<br>◯ 6<br>◯ 7<br>◯ 8<br>◯ 9<br>◯ 10
On a scale of 0-10, how would you rate your overall level of comfort in PLACING A NEW STERILE DRESSING AROUND THE LINE after a central line (Broviac) repair procedure	◯ 0<br>◯ 1<br>◯ 2<br>◯ 3<br>◯ 4<br>◯ 5<br>◯ 6<br>◯ 7<br>◯ 8<br>◯ 9<br>◯ 10
