# Supplementary material for: Trainee educational curriculum to standardize central venous catheter repair
Source: BMC Med Educ. 2023 Dec 19;23:978. doi: 10.1186/s12909-023-04977-9 (PMC10731855; doi:10.1186/s12909-023-04977-9)
Supplement: Supplementary file 3 — Supplementary Material 3: Central venous catheter (CVC) repair policy [file 12909_2023_4977_MOESM3_ESM.pdf]

Central Venous Catheter (CVC) Repair Policy

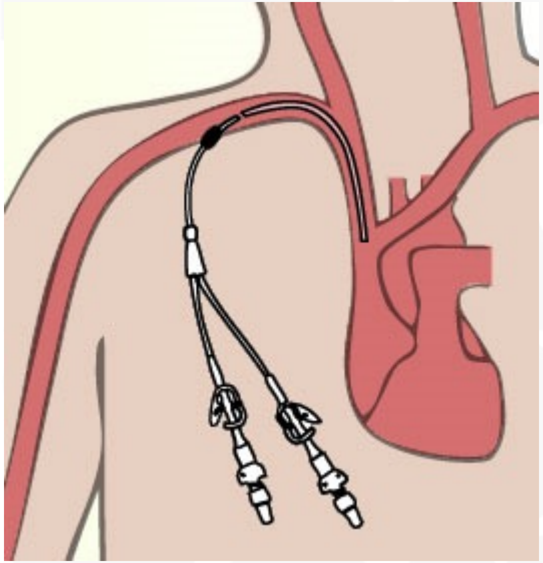

Children's Hospital Colorado
Here, it's different.™

Affiliated with
University of Colorado
Anschutz Medical Campus

Who Can Repair/Qualifications Needed

- Surgery: Attending Physician, Fellow, Resident, and APPs with adequate training
- Adequate training defined as being precepted on a minimum of 3 competent repairs prior to autonomy

Who Can Repair/Qualifications Needed

Indications for Repair Versus Replacement

- Aim to repair within 24 hours of line malfunction.
- Repair criteria: Complete dissection, tear, hole, separation, or ballooning. Must have at least 5cm undamaged catheter remaining distal to insertion site. If repairing only one lumen of a double lumen, must have at least 2.5cm of undamaged catheter distal to bifurcation.
- Existing Current line can be repaired up to three times.
- Line removal +/- replacement criteria: Line will need to be removed and replaced if evidence of soft tissue infection, existing line has been repaired three times, or break occurs <5cm from insertion site.

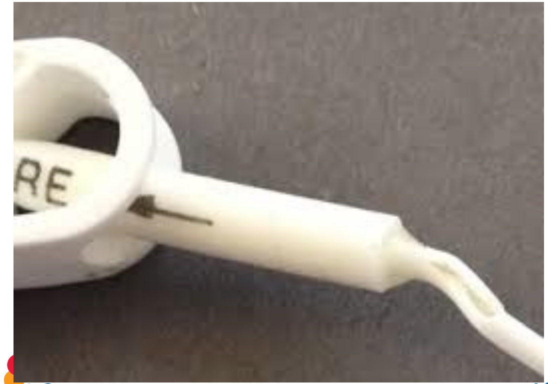

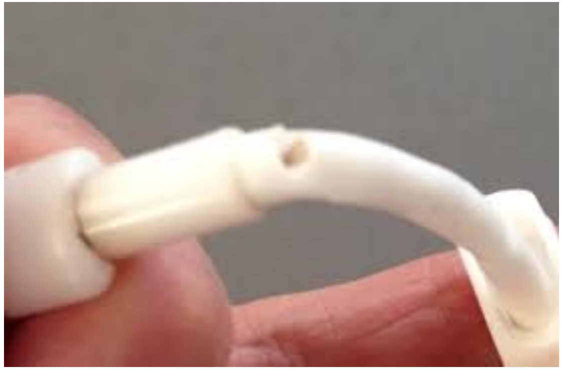

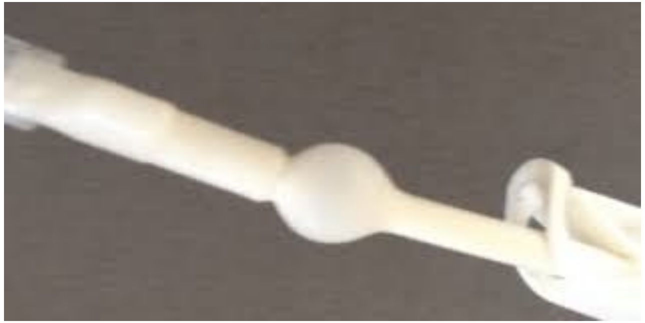

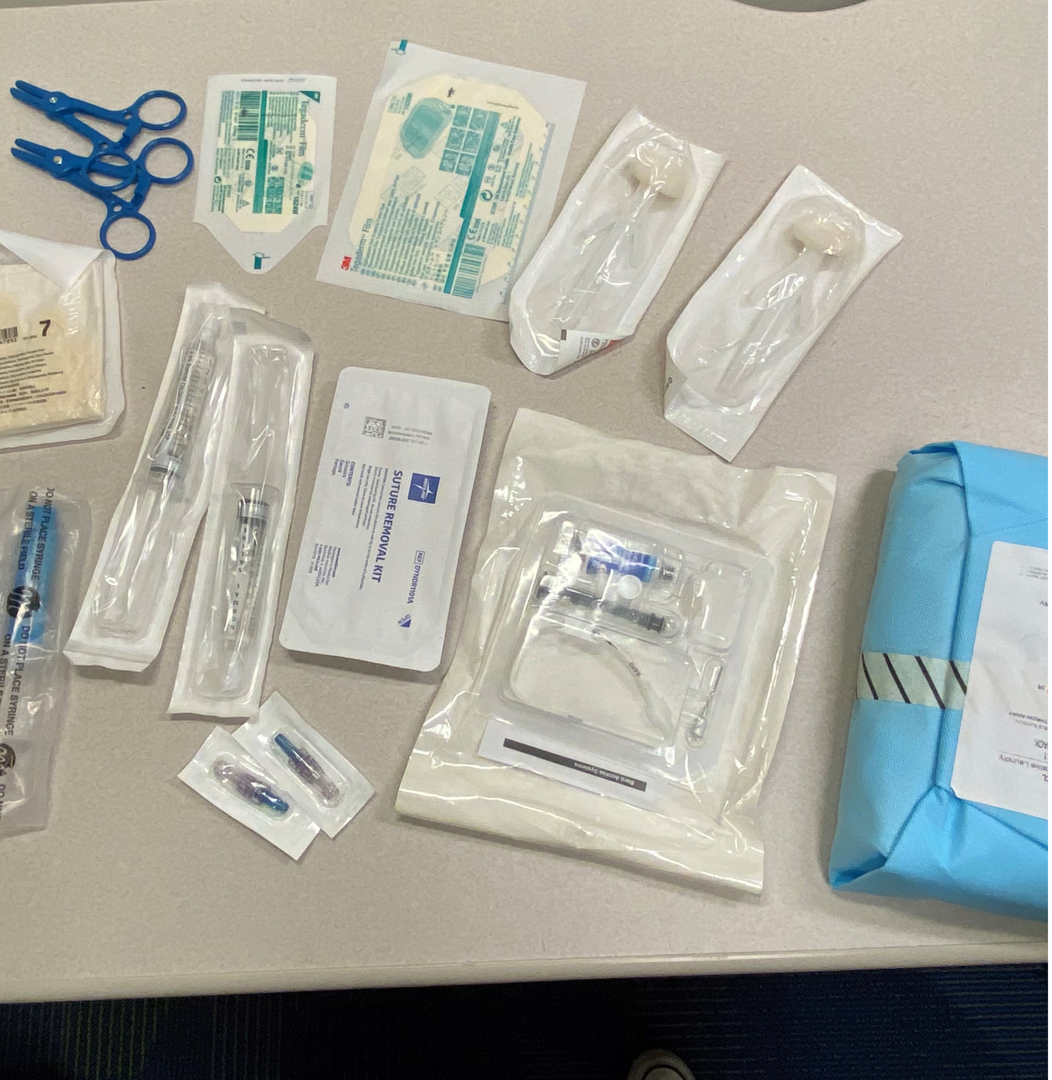

Supplies Needed

Sterile CVC repair kit

Sterile suture removal kit

Masks for all individuals in the room (including patient)

1 pack Sterile gloves

4 pack sterile towels (can be obtained from OR)

Large (3mL) ChloraPrep (2) *If patient has allergy, will need to use iodine sticks

Blue, atraumatic clamps (2)

Needleless connector (Buff Cap) (2)

Transparent Securement dressing in Medium and Small sizes (Tegaderm or Sorbaview per home regimen)

Sterile Saline Flush (2)

1:10 Heparin or Ethanol Flush per home regimen (not sterile)

To obtain these supplies, place an order for “nursing communication.” Within this order, use the dot-phrase of “BROVIACSUPPLIES”

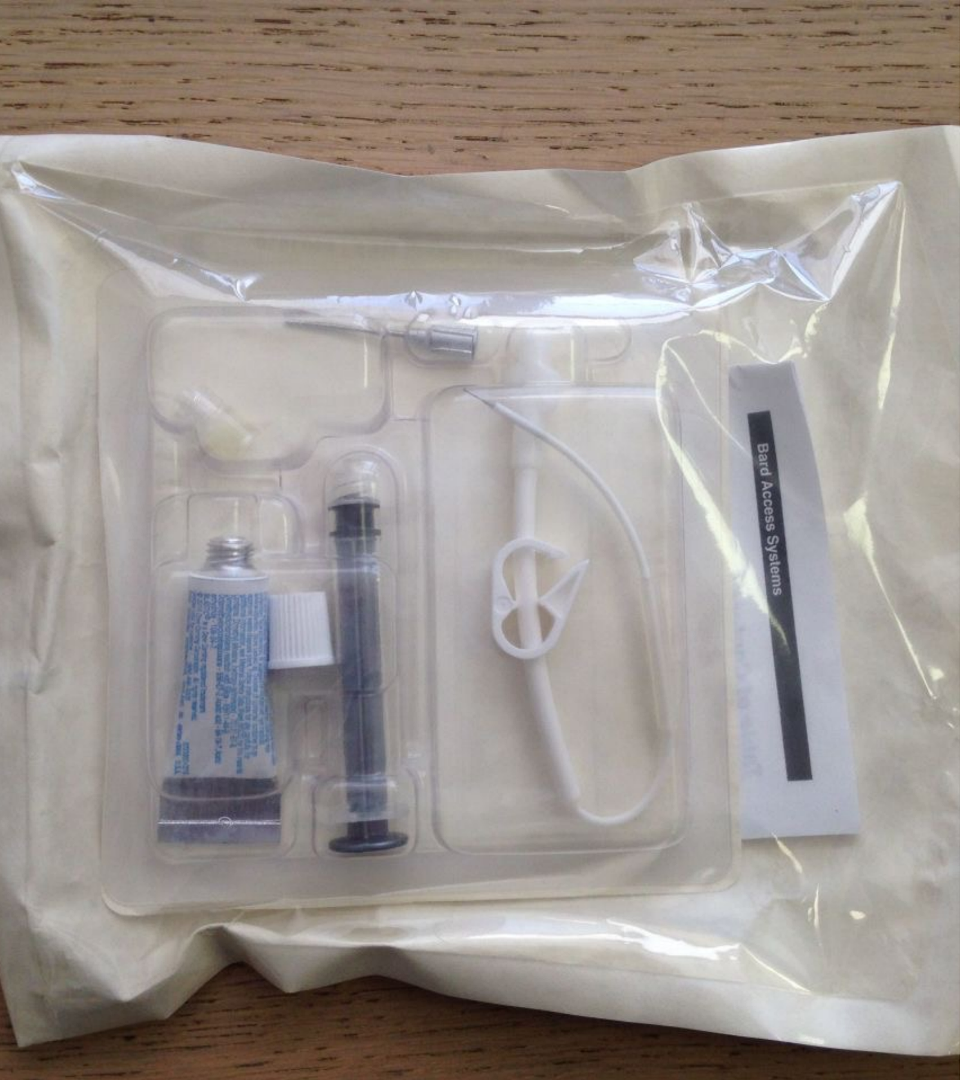

Order Codes for Repair Kits

Name	Epic Code
Hickman Repair Ext Seg 7Fr	190660
Hickman Repair Red Leg 9Fr	190659
Hickman Repair White Leg 9Fr	190658
Broviac Repair Kit 4.2	190638
Broviac Repair Kit 9Fr Dbl Lumen	190632
Broviac Repair Kit 10Fr Dbl Lumen	190633

Order 9Fr (Red or White Leg) if repairing just one lumen for 7,9, 10Fr Double Lumens

Documentation

ENSURE CLASSIFIED AS PROCEDURE NOTE

- Title of Procedure: Central Venous Catheter Repair
- Date Performed
- Performed by
- Assistants
- Indications
- Consent
- Procedure Technique
- Complications

- Can use dot phrase of "BROVIACPROCEDURE"

Teaching for Caregivers

- Do not use line for at least 4 hours, ideally closer to 24 hours.
- The joint will not achieve full mechanical strength for 48 hours.
- Provide family with number for Surgery Outpatient RN to field further inquiries, should they arise at (clinic RN number)
- The number for Pediatric Surgery Clinic is (general clinic number)

Children's Hospital Colorado
Here, it's different.

Affiliated with
University of Colorado
Anschutz Medical Campus

Troubleshooting

- Inability to aspirate blood or flush recently repaired line
    - Can wait four hours and attempt to flush again with saline
    - Can instill thrombolytic agent
    - Last resort is to attempt second CVC repair
